# Pediatric Weight Management Through mHealth Compared to Face-to-Face Care: Cost Analysis of a Randomized Control Trial

**DOI:** 10.2196/31621

**Published:** 2021-09-14

**Authors:** Louise Tully, Jan Sorensen, Grace O'Malley

**Affiliations:** 1 Obesity Research and Care Group, Division of Population Health Sciences School of Physiotherapy Royal College of Surgeons in Ireland, University of Medicine and Health Sciences Dublin Ireland; 2 Healthcare Outcomes Research Centre Royal College of Surgeons in Ireland, University of Medicine and Health Sciences Dublin Ireland; 3 W82GO Child and Adolescent Weight Management Service Children's Health Ireland at Temple Street Dublin Ireland

**Keywords:** childhood obesity, pediatric weight management, economic evaluation, digital health, telemedicine, mHealth

## Abstract

**Background:**

Mobile health (mHealth) may improve pediatric weight management capacity and the geographical reach of services, and overcome barriers to attending physical appointments using ubiquitous devices such as smartphones and tablets. This field remains an emerging research area with some evidence of its effectiveness; however, there is a scarcity of literature describing economic evaluations of mHealth interventions.

**Objective:**

We aimed to assess the economic viability of using an mHealth approach as an alternative to standard multidisciplinary care by evaluating the direct costs incurred within treatment arms during a noninferiority randomized controlled trial (RCT).

**Methods:**

A digitally delivered (via a smartphone app) maintenance phase of a pediatric weight management program was developed iteratively with patients and families using evidence-based approaches. We undertook a microcosting exercise and budget impact analysis to assess the costs of delivery from the perspective of the publicly funded health care system. Resource use was analyzed alongside the RCT, and we estimated the costs associated with the staff time and resources for service delivery per participant.

**Results:**

In total, 109 adolescents participated in the trial, and 84 participants completed the trial (25 withdrew from the trial). We estimated the mean direct cost per adolescent attending usual care at €142 (SD 23.7), whereas the cost per adolescent in the mHealth group was €722 (SD 221.1), with variations depending on the number of weeks of treatment completion. The conversion rate for the reference year 2013 was $1=€0.7525. The costs incurred for those who withdrew from the study ranged from €35 to €681, depending on the point of dropout and study arm. The main driver of the costs in the mHealth arm was the need for health professional monitoring and support for patients on a weekly basis. The budget impact for offering the mHealth intervention to all newly referred patients in a 1-year period was estimated at €59,046 using the assessed approach.

**Conclusions:**

This mHealth approach was substantially more expensive than usual care, although modifications to the intervention may offer opportunities to reduce the mHealth costs. The need for monitoring and support from health care professionals (HCPs) was not eliminated using this delivery model. Further research is needed to explore the cost-effectiveness and economic impact on families and from a wider societal perspective.

**Trial Registration:**

ClinicalTrials.gov NCT01804855; https://clinicaltrials.gov/ct2/show/NCT01804855

## Introduction

### Digital Delivery of Pediatric Weight Management

Mobile health (mHealth), a subcategory of telemedicine whereby clinical care is provided via mobile devices, for weight management in pediatric populations with clinical obesity is an emerging field [1]. For excess adiposity in childhood, family-orientated multidisciplinary weight management, consisting of nutrition and physical activity support with integration of evidence-based behavior-change techniques, is recommended as the cornerstone of treatment [2-4]. There is evidence that telemedicine interventions can support self-management of nutrition and physical activity in children and adolescents [5]; however, there is a scarcity of studies focusing on the economic evaluations of such interventions, particularly for mHealth interventions developed to incorporate evidence-based approaches [1,5,6].

The use of mHealth may improve capacity in terms of delivering health care with a wider geographical reach and may overcome barriers to attending physical appointments experienced by some families using ubiquitous devices such as smartphones and tablets. During the COVID-19 pandemic, technology facilitated alternative modes of delivery for weight management services, which, for many people, meant avoiding long periods of time without professional weight management support [7]. In the long term, such interventions could also expand capacity to areas where long waiting lists, geographical constraints, and staff shortages impose barriers to accessing care.

### Study Rationale

To inform decisions about implementation of mHealth, it is necessary to demonstrate “value for money” in addition to clinical effectiveness for novel treatments and health technologies [8]. Previous studies on mHealth applications for self-management in adult populations have shown promise for potential cost savings [9,10]. However, economic evaluations of telemedicine interventions present methodological challenges in ensuring that the true costs of digital services and their reach are captured in a systematic way that is comparable to face-to-face care [11]. This challenge is compounded by efforts to simultaneously account for the rapid evolution of technology and its effect on resource use and availability; in contrast, the research process (including trial design, implementation, analysis, dissemination, and policy implications) can take many years.

A Tier 3 accredited center of excellence (European Association for the Study of Obesity Centre for Obesity Management) [12] consisting of a multidisciplinary weight management service (the W82GO service) is available for children and adolescents with obesity in Children’s Health Ireland at Temple Street, an urban tertiary care pediatric hospital in the Republic of Ireland [13]. Clinical appointments are either delivered as part of group or one-to-one interventions depending on the needs of the child or adolescent and the preferences of the family. A pilot randomized controlled trial (RCT) [13] tested the clinical effectiveness of a bespoke evidence-based mHealth platform (Android app, clinical portal, and backend database) as an alternative to usual care for the maintenance phase of weight management (3 face-to-face booster sessions over 46 weeks) using a noninferiority design with adolescents. The change in the BMI standardized deviation score (BMI-SDS) was assessed as the primary outcome. The platform was designed using a participatory approach with the intended end users (adolescents with obesity and their parents) [14,15]. The findings of the RCT suggested that substituting face-to-face maintenance care with the mHealth intervention did not adversely affect the change in the primary outcome (BMI-SDS) of the overall treatment. Although study attrition was substantial and was similar to other pediatric trials [16], there was insufficient power to statistically confirm noninferiority [17]. As a result of this, and in addition to high levels of missing secondary outcome data including health-related quality-of-life data, a full cost-effectiveness analysis was not possible despite conducting a clinical trial.

### Study Aim

We aimed to assess the direct costs of delivering the mHealth intervention to participants in the trial relative to usual care participants to inform future designs of mHealth trials to assess effectiveness and cost-effectiveness within this population as well as contribute to the evidence base for the economic viability of integrating mHealth into pediatric weight management services in future.

## Methods

### Design

The pilot noninferiority RCT was approved by the ethics committee of Children’s Health Ireland at Temple Street (reference number 11–033; ClinicalTrials.gov trial registration: NCT01804855). We undertook a microcosting analysis to assess and compare the costs of treatment groups participating in the RCT for pediatric weight management, namely usual care versus mHealth delivered using the “Reactivate” system. We also carried out a budget impact analysis for a 12-month period.

### Sample Size and Recruitment

The null hypothesis in the trial protocol was that the mHealth intervention would have a positive effect on change in the BMI-SDS but that this change will be inferior to that observed in usual care. Based on a reduction of 0.21 in the BMI-SDS at 12 months, an SD of 0.24 in the usual care group, and a noninferiority limit of 0.12, the sample size at 80% power was calculated to be 50 per group or 100 in total. To allow for expected attrition, the target recruitment sample size was 134 [13]. Eligible trial participants were recruited from the W82GO Child and Adolescent Weight Management Service, which is the only dedicated Tier 3 service for children and adolescents with obesity in the Republic of Ireland. All new adolescent referrals made to the service by a pediatrician were screened against the inclusion/exclusion criteria. Those eligible were invited to participate in the study following the consideration of the study by their parents and upon receipt of parental consent and adolescent assent forms.

In total, 109 adolescent participants with clinical obesity (40 boys, 69 girls) were recruited through the W82GO service and received phase 1 of the treatment face to face before being randomized to receive the maintenance phase (phase 2) of treatment either through usual care (three additional face-to-face booster sessions with the multidisciplinary team either through one-to-one sessions or group sessions) or remotely via the mHealth app (Reactivate) [13].

### Data Collection

Participant data including trial group data, whether they commenced one-to-one or group treatment, the number of sessions attended, and records of treatment completion or withdrawal stages, were collected during the trial and used for this analysis to ascertain variations in costs per patient. Cost data were obtained from multiple sources. For face-to-face maintenance sessions, we used a time-driven activity-based microcosting method [18] to capture the direct costs associated with the face-to-face time of health care professionals (HCPs) with patients. We also included administrative time associated with appointment preparation. We interviewed personnel to map workflow processes associated with usual care to accurately assess the unit costs of program appointments and dropout/nonattendance costs. A record of the trial costs was maintained by the principal investigator, and it included invoices received for contracted mHealth service delivery, the related expenses, and the time allocated for checking in, monitoring, and processing participants. During baseline data collection, parents/carers were asked to provide details of their annual income, current occupation, the make and model of their car (if any), mode of transport, and distance traveled to attend hospital appointments.

### Cost Analysis

We carried out our cost analysis based on the detailed unit costs for providing care to both study groups from the perspective of the publicly funded health care system. We undertook the cost analysis under pragmatic “real-world” conditions and their cost implications (ie, estimates of implementing the intervention outside of a research trial) [19], as the trial costs included additional expenses that would not represent the cost of telemedicine if provided as part of usual care (eg, provision of smartphones and mobile data packages to trial participants). We calculated the cost of staff time according to local guidance [20-22], adjusting for pay-related social insurance, pension contributions, annual leave, and overheads. Salaries were calculated using the midpoints from the salary scales for the trial period [23]. For the cost comparison assessment, we also included equipment frequently used for clinical appointments. The unit costs and breakdown of these are shown in Multimedia Appendix 1. Variations in the costs allocated to individuals were based on their treatment group, the completion status, and the number of weeks/sessions completed.

We also undertook a budget impact analysis to assess the cost of providing the mHealth intervention to all eligible adolescents (new referrals) over a 12-month period. In the sensitivity analysis, we evaluated cost assumptions by changing the base case parameters, such as the annual cost of software maintenance, equipment, and variations in the time spent by HCPs in monitoring and supporting adolescents in the mHealth arm. We also examined the impact on the cost per adolescent by changing the optimum treatment cohort size by varying the annual number of users.

We assessed the costs incurred by families based on prospectively collected trial data, but these were not included in the main cost comparison owing to high levels of missing and incomplete data. Therefore, this study considered only the 12-month costs incurred by the publicly funded health care system.

## Results

**Figure 1:**
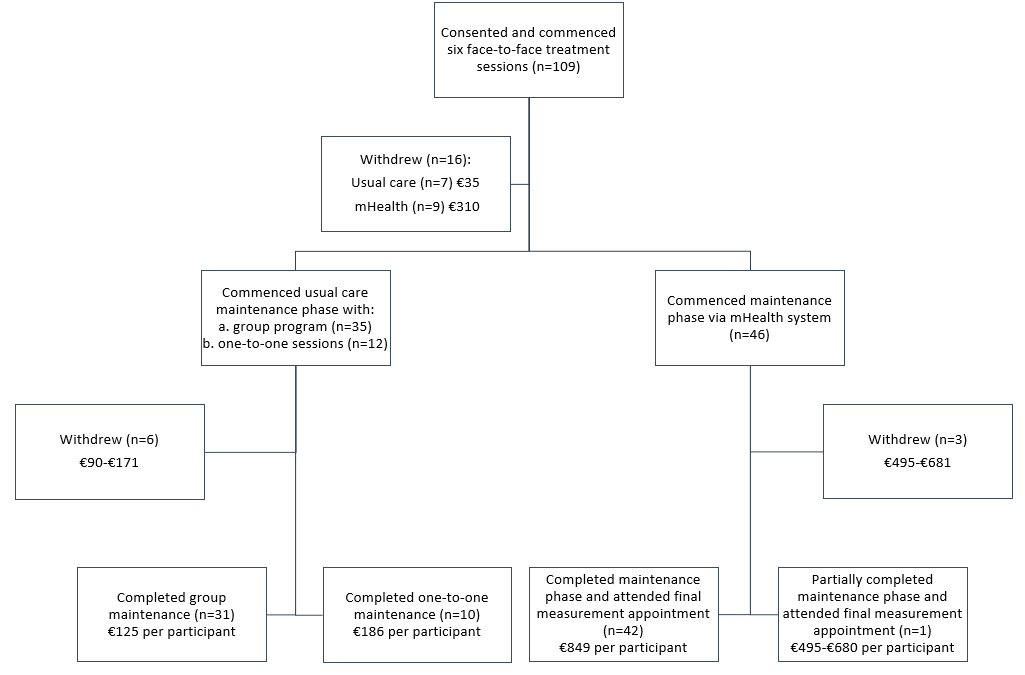
Trial allocation and completion among participants with base case cost estimates. mHealth: mobile health.

In total, 109 adolescents and their families provided consent for participation in the trial; only 84 participants completed the trial as 25 adolescents withdrew from the study (13 from the usual care group and 12 from the mHealth group) after allocation, as shown in Figure 1.

### Health Care System Perspective Costs

The conversion rate for the reference year 2013 was $1=€0.7525. We estimated the mean direct cost per adolescent who completed one-to-one usual care in the maintenance phase of treatment at €186 for all three sessions, as shown in Table 1. For an adolescent who participated in group maintenance sessions, the cost was estimated at €125 (assuming a maximum capacity of 15 families per group). Withdrawal or partial completion costs ranged from €35 to €171 per adolescent for one-to-one sessions depending on the number of sessions missed; withdrawal costs for those in the group treatment were estimated at €153, as their place in the group was lost and could not be filled by another patient. For adolescents who were randomized to use the mHealth system and who completed the program, the mean cost per adolescent was estimated at €849 (based on the intention-to-treat cost divided over all the adolescents allocated to the mHealth arm; n=55). Withdrawal from or partial completion of the mHealth intervention was estimated to cost €310 to €680, depending on when the participant dropped out (see Multimedia Appendix 1).

**Table 1:** Cost per adolescent by treatment group.

Treatment group	Estimated direct cost per participant, mean (SD)
Usual care (one-to-one program)	€176.58 (22.41)
Usual care (group program)	€132.52 (12.18)
mHealth^a^	€722.36 (221.07)

^a^mHealth: mobile health.

Accounting for partial completion and attrition costs, the mean cost incurred for those in the usual care arm was €142 (SD 23.7) (group participants: mean €133, SD 12.2; one-to-one participants: mean €177, SD 22.4). The mean cost for those randomized to use mHealth was estimated to be €722 (SD 221).

The costs for the design and development of the mHealth service (website and app domain name registration and hosting, videography, iconography, device updates for firmware, app development, maintenance costs, and cloud hosting) were independent of the number of users. The sensitivity analysis showed that the main driver of costs for the mHealth group was the HCP time spent managing the mHealth service arm of the trial (platform administration, individualized care plans, providing feedback, troubleshooting, checking in). This was estimated to be approximately 12 hours per adolescent over 46 weeks (approximately 15 minutes per adolescent per week) during the trial. Sensitivity analysis showed that this would need to be reduced to 1.5 hours (2 minutes per adolescent per week), with the number of users increased to 160 before the cost per person would match that of one-to-one, in-person care (€186 per participant). Further, we tested our assumptions around the estimated costs of software maintenance and data storage costs per annum through increasing these by 10%, and this increased the cost per adolescent for the mHealth arm (n=55) by €10, which became negligible once extrapolated to large numbers of users and had a negligible impact on the cost comparison with usual care.

### Budget Impact

Using the cost per adolescent who completed the mHealth intervention and considering the capacity of the weight management service to be 120 new patient referrals per year, we estimated the budget impact of offering the maintenance phase of treatment to all eligible adolescents (BMI ≥98th centile) face to face instead of using mHealth, from the perspective of the health care system. Offering phase 2 of the face-to-face treatment to each eligible adolescent with obesity using the base case has a direct cost of approximately €19,074, whereas the mHealth service would cost €78,120 (excluding app development costs). As such, the direct budget impact of replacing face-to-face maintenance treatment and offering the mHealth intervention to all eligible adolescents in one year would be €59,046, without accounting for potential cost and time savings to be gained by offering mHealth care only.

### Family Perspective

Of the families who took part in the trial, 65% (71/109) provided details about their travel, work, and school arrangements for attending clinical appointments. Further, 17% (19/109) of the families used public transport, at a mean cost of €7 per hospital visit (range €1-€41), whereas 33% (36/109) families drove an average of 17.7 km (range 2-64 km) to their in-person appointments, costing approximately €11 each way (based on a previous study estimating the cost as €0.62 per kilometer including running costs and depreciation [24] plus a €3.10 hourly parking fee). In addition, 4 out of 109 families (4%) took a taxi, with a mean cost of €12 each way (range €5-€15). Using the data provided, the mean cost of travel to and from appointments per adolescent was €18 per visit (€54 for the full face-to-face maintenance phase).

Furthermore, 27% (29/109) of the adolescents had missed school for their appointment on the day of clinic, with an average of 3 hours missed (ranging from 20 minutes to the full school day). As for parents, 21% (22/109) reported that they needed to take time off from work to attend their child’s appointment. Among these 22 parents, 7 needed a full day off and 11 required closer to half a day off; the others did not provide details. Of those who required time off, 18 parents reported their annual income, with 7 earning less than €15,000 per annum and 3 others earning less than €25,000 per annum. In addition, 4 parents earned more than €40,000 per annum and 4 did not report their income. The mean daily salary (adjusted to the whole time equivalent) per parent who provided details of their income was €122 (median €100).

## Discussion

### Principal Findings

This study assessed the treatment costs based on trial data from a pragmatic noninferiority pilot RCT. There was a 23% attrition rate for the trial (25/109); however, this is broadly in line with pediatric RCTs [25] and weight management interventions in general [16], where dropouts are common owing to the intensive nature of these interventions.

The results show that this mHealth intervention, developed using evidence-based approaches, is associated with higher health care costs than face-to-face pediatric weight management. The design of the trial was such that all adolescents attended face-to-face treatment before randomization to either the digital or face-to-face maintenance phase; therefore, this partially digital intervention arm incurred appointment and mHealth costs. The sensitivity analysis results demonstrated that if rolled out to a larger number of users, the main driver of the costs for the mHealth arm is the staff cost related to HCP monitoring and support on a weekly basis. If the mHealth service were to be automated, it could be to reduce these costs; however, further studies would be required to explore the clinical impact of delivering the mHealth service to this clinical population with inputs from less-experienced clinical staff or via increased automation and the associated ethical considerations.

### Comparison With Prior Work

Our finding that staff costs are the most sensitive drivers of the overall cost has been shown in economic evaluations of mHealth in other fields, including care after pregnancy termination [26]. We also found that substantial costs were incurred by the families, but we were unable to fully explore the costs from the perspective of parents and families owing to incomplete data collection. However, this is an important consideration for future research, as assessing ways to reduce inequalities that may be exacerbated by the burden of attending face-to-face appointments is crucial. It is important to explore ways to collect cost data from families, which does not substantially add to the burden of participating in research.

Previous studies have demonstrated that families who live further from clinics, or for whom travel to in-person appointments is more burdensome or complex, tend to view telemedicine more favorably [27]. Despite this, most published economic evaluations of telemedicine consider the perspectives of only the health care service/provider, as shown for cardiovascular disease management [28], obesity prevention [6], and eHealth more broadly [29]. It is important for researchers to assess delivery costs for future evaluations of digitally delivered pediatric weight management to build an evidence base for this population with unique care needs [30]. It is also vital that economic evaluations adopt a societal perspective to capture costs apart from direct health system costs. This has been recommended for mHealth in caring for the elderly as well [31]. The financial strain on parents/caregivers is a documented barrier for pediatric chronic disease management [32] and access to childhood obesity treatment [33], particularly in rural communities [34]. Nelson and colleagues also reported that a telerehabilitation program was not cost-effective for patients recovering from hip replacements but that the reduced burden on patients and caregivers was notable [35]. Clinical pediatric populations are comparable in that patient and caregiver time as well as travel are required for appointments. However, digital interventions may also incur further costs for families, some of which we did not capture, such as internet and phone bills that were covered within the research budget but would add to the burden on families. This highlights the need for further cost studies incorporating a wider perspective that is not limited to the health care provider.

In general, the economic evidence for mHealth is mixed [36]. It is an emerging field, and much of the work to date has evaluated mHealth for health promotion or in the self-management of chronic conditions to prevent the need for health service usage. Such studies are not directly comparable with this trial that evaluated an evidence-based adolescent obesity intervention requiring consistent appointment attendance with an obesity intervention delivered via an mHealth platform as a remote alternative. The body of work associated with the development and testing of the Reactivate mHealth system [13-15,17] has provided novel evidence for the feasibility of using mHealth for pediatric weight management with transparent accounts of the limitations identified, including those relating to the collection of cost data, which will be valuable for informing the design of future robust trials with this vulnerable and complex population.

A recent scoping review [37] on the use of eHealth in diabetes care highlighted critical issues such as staff training, monitoring, technological infrastructure support and maintenance, and how these differ by setting and intervention. Although the mHealth intervention that we evaluated for the maintenance phase of the treatment did not prove economically viable in its prototype form, our results point to design and development aspects where amendments may produce cost savings. The need for 15 minutes of HCP time per participant per week may be a modifiable intervention component. Our study assessed the costs based on the time spent by a senior registered pediatric physiotherapist; however, the option of a more junior staff member managing the mHealth intervention may be feasible, or there may be scope for automating some of the tasks, such as feedback on engagement with the app.

Input from families and HCPs involved in the service could further help identify the acceptability of such modifications. Further, the option of offering both phases of treatment via the mHealth intervention may present a more economically attractive alternative to face-to-face treatment although their clinical effectiveness is unknown. Exploring how this option might suit patients with less complex obesity and fewer complications or comorbidities may also yield evidence for its appropriateness. The acceptability of receiving only remote care for adolescent obesity is also unknown; however, when the mHealth trial was being designed, most families specified a preference for some face-to-face care. This was considered during the design of the pilot RCT. More recently, during the COVID-19 pandemic, up to 40% of families refused virtual appointments from the Child and Adolescent Weight Management Service and preferred to wait longer for face-to-face care. Notwithstanding the preferences of families who are already engaged in treatment, there may be scope to increase access to care through using the mHealth platform with families whose access to evidence-based obesity treatment is limited (eg, children and adolescents who live in rural areas, those who may age out of eligibility for pediatric health care, or those who have no local pediatric obesity treatment services). It may also be possible to achieve cost savings by providing earlier access to treatment via the mHealth platform to adolescents in the community setting and negate the need to join a waiting list for a Tier 3 obesity service. Earlier interventions can reduce or prevent obesity-related complications; given the promising preliminary data on the clinical effectiveness of the mHealth system [17], offering such care to adolescents may mitigate the health effects of obesity at a crucial time during their development.

### Limitations

This study had several strengths and limitations. Assessing the costs incurred by both treatment arms alongside a pragmatic pilot RCT was an important strength of the study, as it reflected the actual costs of delivery in a real-world clinical setting and allowed assumptions that were underpinned by clinical experiences. The microcosting analysis also enabled detailed and accurate direct costing for usual care within the pediatric weight management service. However, the study did not meet the target recruitment number within the available time period, and coupled with the attrition rate, this led to insufficient power for demonstrating statistically significant noninferiority. In addition, low response rates for health-related quality-of-life measures used contributed to the decision of undertaking only a direct cost comparison. As a result, our cost analysis does not provide a full economic evaluation. Further, although it was the only treatment center available nationwide, we acknowledge the limited external validity of our findings given the recruitment through a single center for obesity management. Cost was also not a prespecified outcome for this trial and this study was undertaken as an exploratory analysis after completion of the trial.

Nonetheless, it is important to provide transparent accounts of studies undertaken to assess mHealth interventions with this clinical population, for whom no previous cost studies have been undertaken. It is especially pertinent to document data to describe the economic viability of mHealth, which is often presumed to be a cost-saving alternative to traditional care [38] given the emphasis on digital interventions within the European digital health strategy [39] compounded by the shift in processes resulting from the COVID-19 pandemic.

In addition, access to treatment for obesity is severely limited in Ireland and elsewhere with only approximately 20% of primary care providers reporting sufficient capacity to offer treatment [40]. Therefore, developing and evaluating mHealth interventions for obesity is a high priority for health services. This preliminary research will allow for improved processes and designs aiming to maximize resources while maintaining clinical effectiveness and acceptability among users.

### Conclusions

Childhood obesity remains a leading concern in public health and health services, and the lifetime societal costs have been shown to be substantial [41]. It is important for researchers and practitioners to find new ways to improve the reach and effectiveness of treatments to ensure equitable care. The analyzed digital approach, implemented for the maintenance phase of weight management, was over four times more expensive to deliver than face-to-face maintenance sessions in a pilot RCT. When implemented outside a clinical trial, this cost is likely to reduce owing to the economics of scale and lower costs associated with technology usage. Our results highlight the importance of conducting further research to explore the cost-effectiveness of evidence-informed mHealth interventions in treating chronic diseases such as obesity across multiple centers.

## Appendix

Multimedia Appendix 1 Development of unit costs.

Multimedia Appendix 2 CONSORT-eHEALTH checklist (V 1.6.1).

## Abbreviations

HCP: health care professional
mHealth: mobile health
RCT: randomized controlled trial
SDS: standardized deviation score
