# Supplementary material for: Pediatric Weight Management Through mHealth Compared to Face-to-Face Care: Cost Analysis of a Randomized Control Trial
Source: JMIR Mhealth Uhealth. 2021 Sep 14;9(9):e31621. doi: 10.2196/31621 (PMC8479601; doi:10.2196/31621)
Supplement: Multimedia Appendix 1 [file mhealth_v9i9e31621_app1.docx]

## Supplementary material: unit costs and sources

## Summary of costs

| **Component** | **Unit** | **Cost** | **Source** |
| --- | --- | --- | --- |
| **Usual care** | | | |
| Direct administrative time (book appointment, send invite, phone parent, print handouts, patient check in) – 20 minutes | Pro rata total staff hourly rate per patient | €32.30 | Micro-costing exercises with + Health Service Executive (HSE) 2013 salary scales + Health Information and Quality Authority (HIQA) guidance for calculating staff time (19-21) |
| Keyworker patient assessment and planning pre appointment (clinical coordinator) – 15 minutes |  | €37.59 |  |
| One-to-one session consultation with health and social care practitioner (HSCP) – 45 minutes |  | €115.95 |  |
| Group session (with three HCPs) – 90 minutes |  | €46.38 |  |
| Equipment (laptop and projector) | Equipment cost per child | €66.60 | Estimated cost of €500 per item |
| **mHealth** | | | |
| HSCP time (monitoring, troubleshooting, checking in, for all children in mHealth arm) – 12 hours per week / 15 minutes per child, over 46 weeks | Pro rata total hourly senior physiotherapist rate per patient | €556.01 | Micro-costing exercises + HSE 2013 salary + HIQA guidance for calculating staff time (19-21) |
| Software development and associated costs (videography, iconography, website and app domain name and hosting, device firmware updates) | Per participant in mHealth arm | €198.60 | Trial expense receipts (converted from USD to Euro using retrospective exchange rate where applicable) |
| Smartphones* | Per device | €162.50 | Trial expense receipts |
| Data packages* | Per participant | €33.90 |  |
| Software maintenance |  | €34.76 | Estimated at 15-20% total development costs (https://www.fiercewireless.com/developer/maintaining-app-critical-to-its-overall-success) |
| Cloud data storage |  | €49.09 | Estimated and converted from USD to Euro (https://buildfire.com/hidden-app-development-costs/) |
| Final measurement appointment (administrator and physiotherapist time) | Pro rata total staff hourly rate per patient | €10.74 | Micro-costing exercises + HSE 2013 salary + HIQA guidance for calculating staff time (19-21) |

**Research cost not included in cost comparison*

## Detailed breakdown of unit costs and sources

*Table S1: Staff hourly rate based on HSE 2013 salary scales and calculated according to local Regulatory Impact Analysis guidelines**

| **Salaries** | **A (mid-point of pay range)** | **B (direct salary cost - A + PRSI)** | **C (B + 4% [imputed pension costs])** | **D (C + 25% [overheads])** | **E - Annual working hours calculation ([249-annual leave entitlement] x 6.95)** | **Hourly rate (annual cost of grade ÷ annual leave calculation)** |
| --- | --- | --- | --- | --- | --- | --- |
| Senior physiotherapist | € 59,208.00  (top of scale) | € 65,750.48 | €68,380.50 | € 85,475.63 | 1543 | **€ 55.40** |
| Clerical officer (administrator - grade 4) | € 34,522.50 | € 38,337.24 | €39,870.73 | € 49,838.41 | 1543 | **€ 32.30** |
| Clerical officer (clinical coordinator grade 7) | € 53,545.00 | € 59,461.72 | €61,840.19 | € 77,300.24 | 1543 | **€ 50.10** |
| Senior Dietitian/Physiotherapist | € 54,578.00 | € 60,608.87 | €63,033.22 | € 78,791.53 | 1529 | **€ 51.53** |

**https://govacc.per.gov.ie/wp-content/uploads/Revised_RIA_Guidelines_June_2009.pdf*

*Table S2: Calculations of costs per participant for usual care (1:1 arm) based on micro-costing activities*

| **Activity breakdown** | **Unit** | **Staff member** | **Hourly rate/**  **unit cost** | **Costs** | **Notes** |
| --- | --- | --- | --- | --- | --- |
| ***One to one booster session, in-person*** | ***Time (mins) / equipment*** | ***-*** | ***-*** | ***Per child, per visit*** | ***Where range of minutes provided, mid-point used*** |
| Book appointment on system and send invite | 8 mins | Administrator (grade 4 clerical officer) | €32.30 | €4.31 |  |
| Phone parents for confirmation (week + day before) | 8 mins | Administrator | €32.30 | €4.31 |  |
| Prepare handouts | 2-4 mins | Administrator | €32.30 | €1.62 |  |
| Pre appointment keyworker planning per patient | 15 mins | Clinical coordinator (grade 7 clerical officer) | €50.10 | €12.53 |  |
| HSCP consultation | 45 mins | Physio/  dietitian | €51.53 | €38.65 |  |
| Total per appointment | - | - | - | €61.94 |  |
| Total cost for maintenance phase of treatment (completer) | - | - | - | **€185.82** | x3 booster sessions |
| Total cost for partial completion: dropped out before phase two | 13 mins +  30 mins | Administrator  + senior physio | €32.30  €55.40 | **€34.70** | Invite/booking preparation admin + 30 minutes follow up and processing for dropout |
| Total cost for partial completion: did not attend after session one of three | 13 mins +  15 mins +  30 mins | Administrator + clinical coordinator + senior physio | €32.30  €50.10  €55.40 | **€109.16** | One booster session @ €61.94 + invitation preparation admin + pre-session keyworker planning + 30 minutes follow up and processing for dropout |
| Total cost for partial completion: dropped out after session one of three | 30 mins | Senior physio | €55.40 | **€89.64** | One booster session @ €61.94  + 30 minutes follow up and processing for dropout |
| Total cost for partial completion: did not attend after session two of three | 13 mins +  15 mins +  30 mins | Administrator + clinical coordinator + senior physio | €32.30  €50.10  €55.40 | **€171.10** | Two booster sessions @ €61.94  + invitation preparation admin  + pre-session keyworker planning + 30 minutes follow up and processing for dropout |
| Total cost for partial completion: dropped out after session two of three | 30 mins | Senior physio | €55.40 | **€151.68** | Two booster sessions @ €61.94 + 30 minutes follow up and processing for dropout |

*Table S3: Calculations of costs per participant for usual care (group arm) based on micro-costing activities*

| **Activity breakdown** | **Unit** | **Staff member** | **Hourly rate/**  **unit cost** | **Costs** | **Notes** |
| --- | --- | --- | --- | --- | --- |
| ***Group booster session, in-person*** | ***Time (mins) / equipment*** | ***-*** | ***-*** | ***Per child, per visit*** | ***Where range of minutes provided, mid-point used*** |
| Laptop | 1 | - | - | €33.30 | Estimated cost of €500 |
| Projector | 1 | - | - | €33.30 | Estimated cost of €500 |
| Booking group appts | 15 mins | Administrator | €32.30 | €8.08 |  |
| Book room | 10-30 mins | Administrator | €32.30 | €10.77 |  |
| Send invites | 60 mins | Administrator | €32.30 | €32.30 |  |
| Phone parents for confirmation (week + day before) | 120 mins | Administrator | €32.30 | €64.60 |  |
| Prepare handouts | 30-60 mins | Administrator | €32.30 | €24.23 |  |
| Pregroup keyworker planning | 225 mins (15 mins per patient) | Clinical coordinator (grade 7 clerical officer) | €50.10 | €187.88 |  |
| Group booster session with three HSCPs | 90 mins | Physio/  dietitian | €51.53 | €231.90 |  |
| Total per appointment |  |  |  | €41.76 |  |
| Total for maintenance phase of treatment (completer) |  |  |  | **€125.27** |  |
| Total cost for maintenance phase (partial completer) | 30 mins | Senior physio | €55.40 | **€152.97** | Full cost incurred as place in group is lost + 30 minutes follow up and processing for dropout |

*Table S4: Calculations of costs per participant in mHealth arm based on micro-costing activities*

| **Activity breakdown** | **Unit** | **Staff member** | **Hourly rate/**  **unit cost** | **Costs** | **Notes** |
| --- | --- | --- | --- | --- | --- |
| ***mHealth*** |  |  |  | ***Per child*** | ***Where range provided, mid-point used*** |
| HSCP time monitoring (checking in, troubleshooting, providing feedback to) app users | Hours (12 per week over 46 weeks) | Senior physio | €30,580.80 | €556.01 |  |
| Software development + related costs | Sum total converted / participants | External contractors | €10,923.00 | €198.60 | Divided between n=55 participants randomised to mHealth |
| Final measurement appointment | Staff time | Administrator, physio | Admin €32.30,  Physio €51.53 | €10.74 | Admin check in 2-6 mins, physio measures 10 mins |
| Software maintenance | 15-20% total development costs |  | €1,911.53 | €34.76 | Source: https://www.fiercewireless.com /developer/maintaining  -app-critical-to-its-overall-success  Divided between n=55 participants randomised to mHealth |
| Data storage | Annual cost estimate |  | $3600 pa total | €49.09 | Source: <https://buildfire.com/hidden-app-development-costs/>  Converted using midpoint of other conversion rates (0.75)  Conversion rate used:  https://www.pound sterlinglive.com/best-exchange-rates/best-us-dollar-to-euro-history-2013 |
| Total cost per user for maintenance phase (completer) |  |  |  | **€849.20** |  |
| Total cost for partial completion: dropped out before phase two | Software development, maintenance and storage per child  Processing dropout | Estimated costs calculated (above)  Senior physio  30 mins | €282.45  €55.40 per hour | **€310.15** | All app development and maintenance costs (excluding weekly staff check-in and monitoring costs)  + 30 minutes follow up and processing for dropout |
| Total cost for partial completion (dropout around 33% through maintenance phase): | Software development, maintenance and storage per child  15 mins per week monitoring + processing dropout | Estimated costs calculated (above)  Senior physio | €282.45  €55.40 per hour | **€495.30** | All app development and maintenance costs  + weekly staff check-in and monitoring costs (x 0.33 = €185.15)  + 30 minutes follow up and processing for dropout |
| Total cost for partial completion (dropout around 67% through maintenance phase): | Software development, maintenance and storage per child  15 mins per week monitoring + processing dropout | Estimated costs calculated (above)  Senior physio | €282.45  €55.40 per hour | **€680.45** | All app development and maintenance costs  + weekly staff check-in and monitoring costs (x 0.66 = €370.31)  + 30 minutes follow up and processing for dropout |
